# Enhanced Synthesis and Diminished Degradation of Hydrogen Sulfide in Experimental Colitis: A Site-Specific, Pro-Resolution Mechanism

**DOI:** 10.1371/journal.pone.0071962

**Published:** 2013-08-05

**Authors:** Kyle L. Flannigan, Jose G. P. Ferraz, Rui Wang, John L. Wallace

**Affiliations:** 1 Farncombe Family Digestive Health Research Institute, McMaster University, Hamilton, Ontario, Canada; 2 Inflammation Research Network, University of Calgary, Calgary, Alberta, Canada; 3 Department of Biology, Lakehead University, Thunder Bay, Ontario, Canada; CWRU/UH Digestive Health Institute, United States of America

## Abstract

Hydrogen sulfide (H_2_S) is produced throughout the gastrointestinal tract, and it contributes to maintenance of mucosal integrity, resolution of inflammation, and repair of damaged tissue. H_2_S synthesis is elevated in inflamed and damaged colonic tissue, but the enzymatic sources of that synthesis are not completely understood. In the present study, the contributions of three enzymatic pathways to colonic H_2_S synthesis were determined, with tissues taken from healthy rats and rats with colitis. The ability of the colonic tissue to inactivate H_2_S was also determined. Colonic tissue from rats with hapten-induced colitis produced significantly more H_2_S than tissue from healthy controls. The largest source of the H_2_S synthesis was the pathway involving cysteine amino transferase and 3-mercaptopyruvate sulfurtransferase (an α-ketoglutarate-dependent pathway). Elevated H_2_S synthesis occurred specifically at sites of mucosal ulceration, and was not related to the extent of granulocyte infiltration into the tissue. Inactivation of H_2_S by colonic tissue occurred rapidly, and was significantly reduced at sites of mucosal ulceration. This correlated with a marked decrease in the expression of sulfide quinone reductase in these regions. Together, the increased production and decreased inactivation of H_2_S at sites of mucosal ulceration would result in higher H_2_S levels at these sites, which promotes of resolution of inflammation and repair of damaged tissue.

## Introduction

Hydrogen sulfide is produced in virtually every organ system in the body [1], [2] and can modulate a variety of physiological processes, including vasodilation [3], neurotransmission [4], nociception [5], [6] and inflammation [7]–[9]. Based on these observations, a number of H_2_S-releasing therapeutic agents are in development for a wide range of disorders [8], [10]–[17]. The importance of H_2_S in the gastrointestinal tract is highlighted by its ability to regulate intestinal smooth muscle function [18], [19] and epithelial secretion [20]–[22], as well as playing important roles in mucosal defence [14], [23]–[26]. H_2_S can enhance ulcer healing [27] and promote the resolution of colitis [25], [28], [29]. In recent years it has become clear that H_2_S is an important substrate for mitochondrial respiration, driving production of adenosine triphosphate, particularly in circumstances of reduced oxygen concentrations [30]. Colonocytes are particularly well adapted for using H_2_S as an energy source [31], [32], and may act as a ‘metabolic barrier’, limiting penetration of bacteria-derived H_2_S into the mucosa [26]. There is also evidence that during hypoxia, mitochondrial H_2_S metabolism may underlie the ability of this gasotransmitter to reduce reactive oxygen metabolite-mediated tissue injury [33]–[35]. Such effects could contribute to the ability of H_2_S to protect the gastrointestinal mucosa from injury and to promote healing [26].

The majority of H_2_S production in mammalian tissue is enzymatically regulated, with the pyridoxal-5′-phosphate (P5P)-dependent enzymes cystathionine-beta-synthase (CBS) and cystathionine-gamma-lyase (CSE) being the most extensively studied [1]. During a bout of hapten-induced colitis in rats, the capacity of colonic tissue to produce H_2_S is substantially increased, in parallel with the degree of mucosal inflammation [25]. The H_2_S produced in this setting exerts significant beneficial effects in terms of reducing inflammation and enhancing healing of the ulcerated tissue [25]. Based on pharmacological studies (i.e., using inhibitors of CBS and CSE), CBS appeared to account for the majority of the observed increase in colonic H_2_S synthesis during colitis [25]. However, administration of inhibitors of both CBS and CSE did not completely abolish colonic H_2_S production, raising the possibility that there may be other sources of H_2_S synthesis in the colon [25]. We previously reported that enteric bacteria did not account for a significant portion of what we have measured as colonic tissue H_2_S synthesis [36].

**Figure 1:**
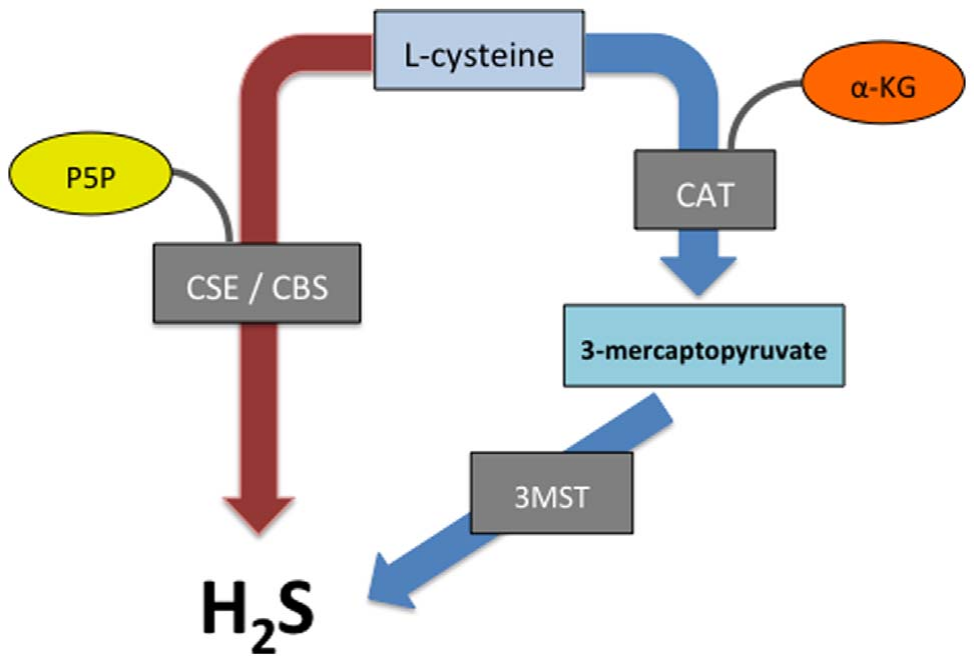
Major pathways of colonic hydrogen sulfide (H_2_S) synthesis. Hydrogen sulfide can be produced from L-cysteine thorugh at least three enzymatic pathways. The pyridoxal-5′-phosphate (P5P)-dependent enzymes, cystathionine-β-synthase (CBS) and cystathionine-Υ-lyase (CSE), can metabolize L-cysteine, resulting in the generation of H_2_S. L-cysteine can also be converted to 3-mercaptopyruvate via the enzyme cysteine aminotransferase (CAT), the activity of which is depending upon the presence of α-ketoglutarate (α-KG). Mercaptopyruvate transferase (3MST), which is largely localized to mitochondria, can metabolize 3-mercaptopyruvate to generate H_2_S.

A third enzymatic pathway for H_2_S synthesis, which does not require P5P as a co-factor, was identified in the brain of CBS-deficient mice by Shibuya et al. [37] (Figure 1). Cysteine aminotransferase (CAT), which requires α-ketoglutarate as a co-factor, converts L-cysteine into 3-mercaptopyruvate, which can then be converted by mercaptopyruvate sulfurtransferase (3MST) to H_2_S and pyruvate [37], [38]. This pathway has also been shown to contribute to H_2_S synthesis in the vascular endothelium of the thoracic aorta 37,38.

In the present study, we examined the contribution of the CAT-3MST pathway and the two P5P-dependent pathways to H_2_S synthesis in the healthy and inflamed colon. We also attempted to determine the relative contributions of the ulcerated versus non-ulcerated colonic mucosa to H_2_S synthesis, and we examined the capacity of these tissues to inactivate H_2_S. Our results demonstrate that there is a site-selective enhancement of H_2_S synthesis, as well as decreased H_2_S inactivation, in the regions of ulceration. Such changes are consistent with an important role of this gasotransmitter in repair of injury and resolution of inflammation.

## Methods and Materials

### Animals

Male, Wistar rats (from Charles River Breeding Farms, St-Constant, QC, Canada) were housed in plastic cages and maintained under controlled temperature (20°C), humidity (60%–70%), and light cycle (12 h∶12 h light-dark). The rats were fed standard laboratory chow and water *ad libitum*. All experimental protocols were approved by the Animal Research Ethics Board at McMaster University, and adhered to the guidelines established by the Canadian Council on Animal Care.

Mice with a targeted disruption of the CSE gene [39] and from age-matched (16 weeks), wild type littermates on the C57BL/6J X 129SvEv background were housed in the animal care facility at Lakehead University (experimental protocols were approved by the Animal Care Committee of Lakehead University). Heterozygous CBS deficient (CBS+/−) mice on the C57BL/6 background were obtained from Jackson Laboratories (Bar Harbor, ME, USA) and bred and maintained in the animal care facility at McMaster University. Homozygous CBS deficient mice (CBS−/−) display significant growth retardation and rarely survive past 5 weeks of age, therefore CBS+/− mice were used [40]. Age-matched (20 weeks), wild type (CBS +/+) littermates were used as controls.

### Induction of colitis

Colitis was induced in conventionally housed rats using dinitrobenzene sulfonic acid (DNBS) [41], a modified version of the original trinitrobenzene sulfonic acid model of colitis [42]. Rats were given 30 mg DNBS intracolonically in 0.5 mL of 50% ethanol. Groups of rats (n≥4) were euthanized 6 h to 28 days after DNBS administration for determination of H_2_S synthesis. Colonic inflammation was assessed by the measurement of myeloperoxidase (MPO) activity, using a method modified [43] from that described by Bradley et al. [44]. MPO is an enzyme found primarily in the azurophilic granules of neutrophils, and has been used extensively as a quantitative index of granulocyte infiltration.

### Separation of colonic mucosa and muscularis

After the rats were euthanized, the colons were excised, opened along the mesenteric border, washed thoroughly to remove fecal material and immediately placed in ice-cold potassium phosphate buffer, pH 8.0 (12% w/v). Sections of the distal colon were visualized under a dissecting microscope and pinned along the edges of the sections with care taken to minimize damage to the tissue. The border between the mucosa and smooth muscle layer was identified, then these two layers were gently separated using forceps, as described by Lowie et al. [45], and immediately snap-frozen in liquid nitrogen (for measurements of H_2_S synthesis) or fixed in neutral-buffered formalin (for histology). In rats that had received DNBS, the mucosal and smooth muscle layers were separated in the same manner, then sections were cut at the border between the ulcer and the immediately adjacent non-ulcerated tissue, yielding separate mucosal and smooth muscle sections from both ulcerated and non-ulcerated areas. The tissues that were fixed in formalin were processed and stained with hematoxylin & eosin for microscopic examination, to confirm separation of the mucosal layer from the smooth muscle.

### Measurement of H_2_S synthesis

The capacity of tissue to produce H_2_S was measured from homogenized tissue in the presence of exogenous substrate and/or inhibitors using a modified version of a previously described zinc-trapping assay [14], [46]. Production of H_2_S via the CAT-3MST pathway was determined using the substrate α-ketoglutarate (α-KG; 100 µM unless otherwise stated) and the competitive CAT inhibitor L-aspartate, and O-carboxymethyl-hydroxylamine hemihydrochloride (CHH; an inhibitor of aminotransferases, including CAT). Addition of the substrate, L-cysteine (10 mM), was necessary for detection of H_2_S synthesis (P5P-dependent or -independent) by colonic tissue. H_2_S synthesis via CSE and CBS required the presence of P5P (2 mM unless otherwise stated), while that via CAT/3MST required α-KG.

### Measurement of H_2_S inactivation

The ability of colonic tissue to inactivate H_2_S, such as through metabolism and/or sequestration, was measured using a modified version of the above-mentioned zinc-trapping assay. Instead of including L-cysteine as a substrate for H_2_S synthesis, vials containing either tissue homogenates or buffer were ‘spiked’ with 33 µL of 30 µM NaHS, the same H_2_S-releasing compound that is used to generate the standard curve for the H_2_S assay. The samples were then incubated for 5–90 min in a 37°C shaking water bath, after which trichloroacetic acid (TCA; 50%; 0.4 mL) was injected into the reaction mixture through the serum cap to halt the reaction. The vials were then transferred to a 50°C shaking water bath for 60 min to allow for the trapping of evolved H_2_S by the zinc acetate. The greater the ability of a tissue to metabolize/sequester H_2_S, the less H_2_S would be trapped in the zinc acetate and subsequently detected in the reaction with FeCl_3_ and N,N-dimethyl-p-phenyline-diamine sulfate salt, which results in methylene blue formation. To determine the recovery of H_2_S in this assay in the absence of enzymatic activity, samples were treated in the same way, except that the TCA was added prior to addition of the NaHS. In this case, the amount of H_2_S recovered 30 min later was ∼80% of that recovered if the NaHS was added to vials not containing any tissue. In addition, in some experiments, potassium cyanide (1 mM) was added to the tissue samples immediately prior to the addition of NaHS to abolish mitochondrial activity and 30 min later the effects on recovery of H_2_S were determined.

### Expression of H_2_S producing and catabolizing enzymes

Western blot analysis was used to determine colonic expression of CBS, CSE, CAT, 3MST, and sulfide quinone reductase (SQR) in samples from rats with colitis and healthy control rats. Colonic tissue was processed and blots were prepared as previously described [27]. Proteins were separated on 4–20% gradient polyacrylamide gels. Rabbit polyclonal anti-CSE (1∶200), anti-CBS (1∶800), anti-CAT (1∶800), anti-3MST (1∶200), and anti-SQR antibodies (1∶500) were used. Enzyme expression was visualized using a secondary anti-rabbit IgG antibody conjugated to horseradish peroxidase (1∶1000) and an enhanced chemiluminescence detection kit on a Chemi-doc gel imaging system (Bio-Rad). The intensity of the bands was determined and analyzed using ImageLab 2.0 software (Bio-Rad). The expression of each enzyme was normalized to the expression of β-actin.

### Materials

Isoflurane was obtained from Abbott Laboratories (Montreal, QC, Canada). 4–20% gradient polyacrylamide gels were purchased from Bio-Rad Laboratories (Mississauga, ON, Canada). Anti-CSE, anti-CAT, and anti-SQR primary antibodies were obtained from Proteintech (Chicago, IL, USA). Anti-CBS and anti-3MST primary antibodies were obtained from Santa Cruz Biotechnology Inc. (Santa Cruz, CA, USA). The secondary anti-rabbit IgG antibody conjugated to horseradish peroxidase was purchased from Invitrogen (Camarillo, CA, USA). The enhanced chemiluminescence detection kit was obtained from GE Healthcare (Baie d'Urfe QC, Canada). All other reagents were purchased from Sigma-Aldrich Co. (Oakville, ON, Canada).

### Statistical analysis

All data are expressed as the mean ± SEM. Groups of data were compared to one another using a one-way analysis of variance and the Neuman-Keuls test. An associated probability of less than 5% was considered significant.

## Results

### Healthy colonic tissue produces H_2_S via the CAT-3MST pathway

**Figure 2:**
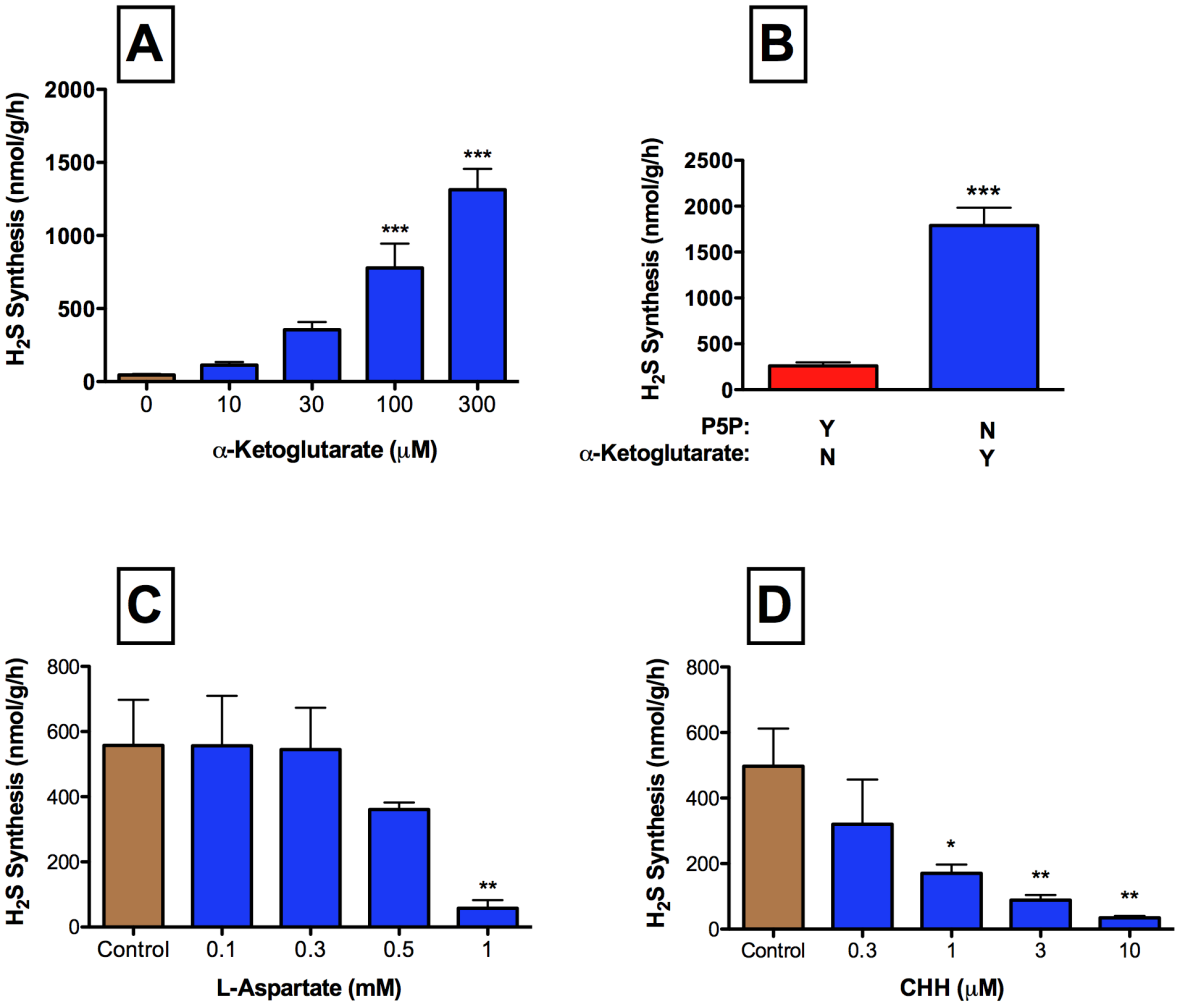
H_2_S synthesis by healthy colonic tissue occurs via multiple pathways. Panel A: In the absence of pyridoxal-5′-phosphate (P5P), α-ketoglutarate increased the production of H_2_S in by colonic tissue in a concentration-dependent manner (***p<0.001 vs. the group with no α-ketoglutarate added). Panel B: Maximal production of H_2_S from L-cysteine by the healthy colon was observed in the presence of 3 mM P5P or 300 µM α-ketoglutarate. The maximal production of H_2_S in the healthy colon in the presence of α-ketoglutarate was markedly higher than the maximal production of H_2_S in the presence of P5P. Panel C: At a concentration of 1 mM, L-aspartate significantly inhibited H_2_S synthesis by healthy colonic tissue. Panel D: CHH (O-carboxymethyl-hydroxylamine hemihydrochloride) concentration-dependently inhibited α-ketoglutarate-dependent colonic H_2_S synthesis. The reactions shown in Panels C and D were carried out in the absence of pyridoxal-5′-phosphate and presence of α-ketoglutarate (100 µM). In the absence of both P5P and α-ketoglutarate there was no detectable H_2_S production. Each bar represents the mean ± SEM of 4–6 rats (*p<0.05, **p<0.01, ***p<0.001 vs. the control group).

Colonic H_2_S synthesis via the CBS/CSE pathways versus the CAT-3MST pathway could be delineated through inclusion or exclusion of the co-factors P5P and α-KG (Figure 1). Healthy rat colonic tissue produced H_2_S in the absence of P5P and the presence of α-KG, consistent with synthesis via the CAT-3MST pathway (Figure 2A). The amounts of H_2_S produced increased with the concentration of α-KG.

To determine the relative contributions of the CBS/CSE versus the CAT-3MST pathways to colonic H_2_S synthesis in rats, we determined the optimal concentrations of co-factors (P5P and α-KG, respectively) for each pathway, by performing concentration-response studies. Maximal production of H_2_S via the CBS/CSE pathways was observed with P5P at 3 mM (not shown), while maximal production of H_2_S via the CAT-3MST pathway was observed with α-KG at 300 µM (Fig. 2A). As shown in figure 2B, under these optimized conditions, the CAT-3MST pathway accounted for about 6-times as much colonic H_2_S synthesis as that produced via the CBS/CSE pathways (p<0.001).

Further evidence for colonic synthesis of H_2_S from the CAT-3MST pathway was provided by studies of inhibitors of this pathway in rats. Both L-aspartate and CHH, which inhibit CAT activity, significantly and concentration-dependently reduced α-KG-dependent H_2_S synthesis by healthy colonic tissue (Figure 2C and D, respectively).

### CSE is the major contributor to P5P-dependent H_2_S synthesis in the healthy colon

**Figure 3:**
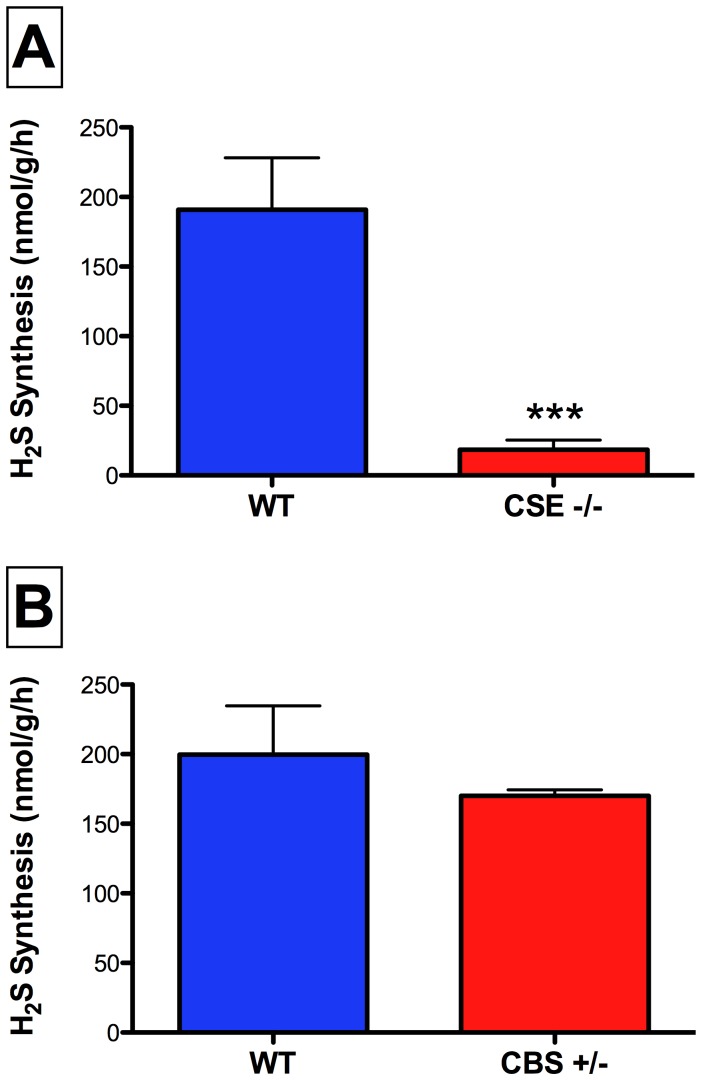
Colonic H_2_S synthesis in CSE-deficient (CSE−/−) and CBS heterozygous (CBS+/−) mice. Panel A: In the presence of P5P, colonic tissue from CSE-deficient mice produced significantly less H_2_S from L-cysteine than tisue from wild type mice (***p<0.001). Panel B: There was no difference in the ability of colonic tissue from CBS heterozygous mice to produce H_2_S from L-cysteine in the presence P5P when compared to colonic tissue from wild type controls. Each bar represents the mean ± SEM of 4–7 mice.

To determine the main enzymes responsible for P5P-dependent H_2_S production in the colon we examined colonic H_2_S production in CSE−/− and CBS+/− mice. Colonic H_2_S synthesis was reduced by ∼90% in CSE-deficient mice as compared to their wild-type littermates (Figure 3A). In contrast, despite having decreased expression of the CBS enzymes, colonic H_2_S production in CBS+/− mice was not significantly different from that in wild type mice (Figure 3B). These data suggest that CSE is the predominant source of P5P-dependent H_2_S synthesis by healthy mouse colon.

### H_2_S is produced via CAT-3MST in inflamed colon

**Figure 4:**
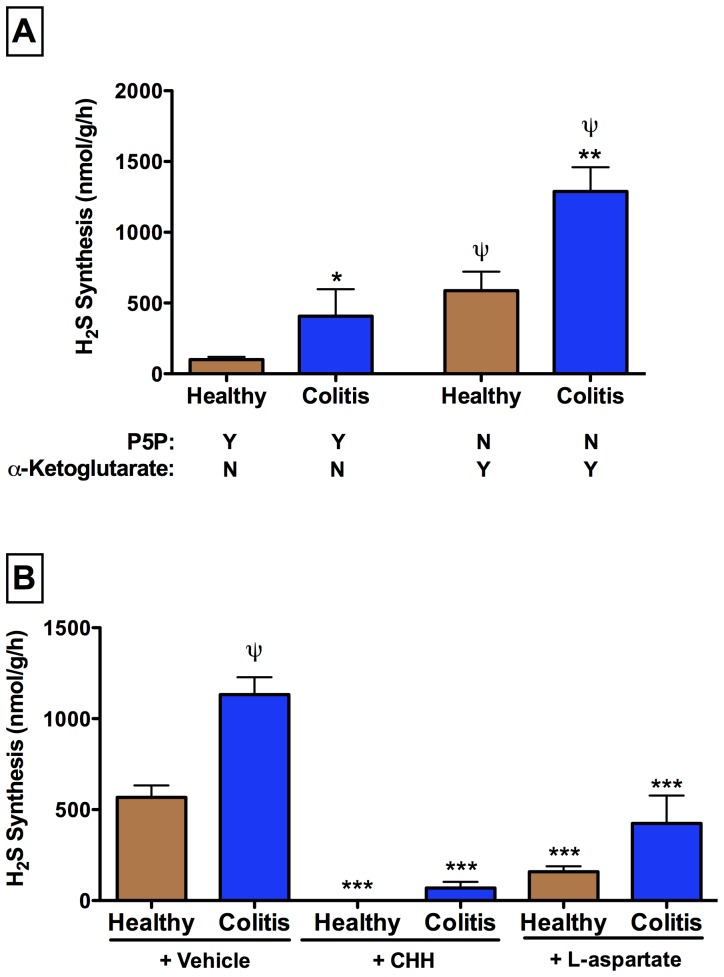
Sources of H_2_S synthesis in the healthy and inflamed rat colon. Panel A: In the presence of P5P and absence of α-ketoglutarate, colonic tissue from rats with colitis (3 days post-DNBS administration) synthesized significantly more H_2_S than that from healthy rats (*p<0.05 vs. corresponding healthy group). Likewise, synthesis of H_2_S by colonic tissue from rats with colitis was significantly greater than that from healthy rats when the assay was performed in the presence of α-ketoglutarate and the absence of P5P. For both healthy rats and rats with colitis, colonic H_2_S synthesis was significantly greater via the α-ketoglutarate-dependent pathway than via the P5P-dependent pathways (^ψ^p<0.05). In the absence of both P5P and α-ketoglutarate there was no detectable H_2_S production. Panel B: α-ketoglutarate-dependent H_2_S synthesis by samples of colonic tissue from healthy rats and rats with colitis (3 days post-DNBS administration) was significantly inhibited by CHH (O-carboxymethyl-hydroxylamine hemihydrochloride) (10 µM) and by L-aspartate (1 mM) (***p<0.001 vs. the corresponding vehicle-treated group; (^ψ^p<0.05) vs. the corresponding healthy group). Each bar represents the mean ± SEM of 4–6 rats.

A pilot time-course study demonstrated that the greatest granulocyte infiltration of the colon occurred at 3 days after DNBS administration (MPO activity in DNBS-treated rats averaged 22.2±4.3 U/mg, versus 4.6±0.5 U/mg in healthy controls; p<0.05). Colonic H_2_S synthesis was significantly elevated in samples from rats with colitis, both through the P5P-dependent pathways and the CAT/3MST pathway (Figure 4A). As in the case for colonic tissue from healthy rats, there was substantially greater H_2_S synthesis from the CAT/3MST pathway in tissue from rats with colitis than there was from the P5P-dependent pathways. The two inhibitors of CAT (CHH and L-aspartate) each substantially reduced α-KG-dependent H_2_S synthesis by inflamed colonic tissue (Figure 4B).

### Ulcerated mucosa is the major site of elevated H_2_S production

**Figure 5:**
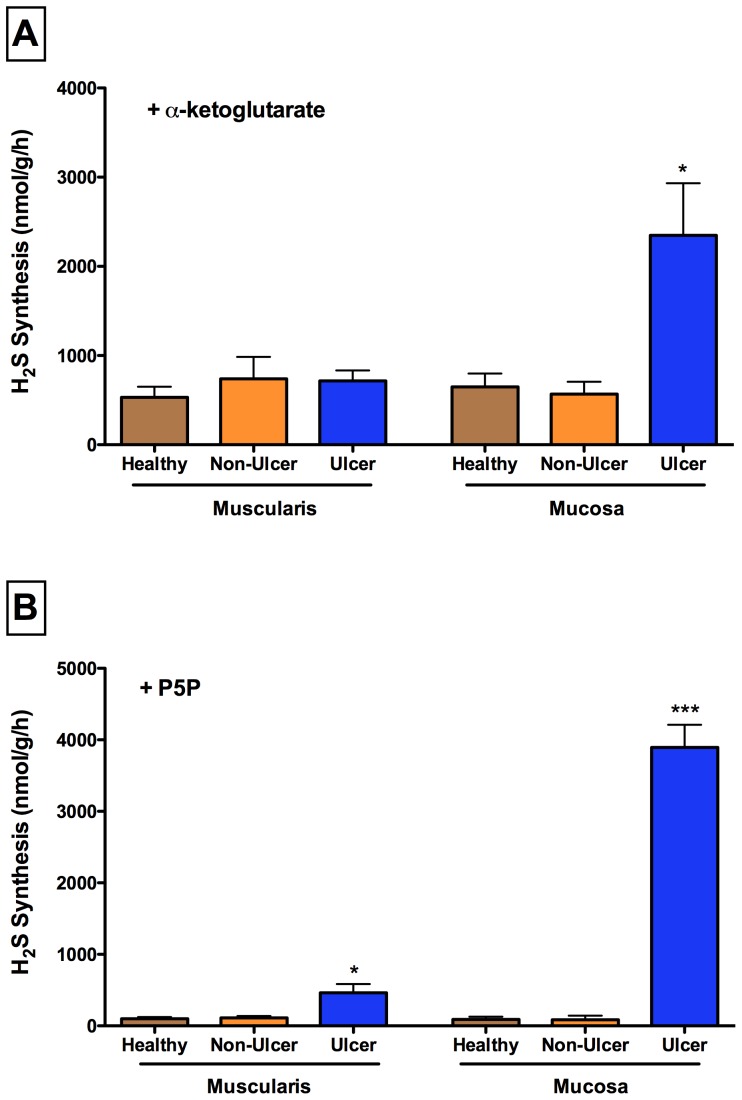
H_2_S synthesis by compartments of healthy and damaged colon. Panel A: The contributions of the mucosa and muscularis layers of the colon to H_2_S synthesis via the α-ketoglutarate-dependent pathway. H_2_S synthesis by the ulcerated mucosa was markedly increased compared to that of adjacent, non-ulcerated mucosaand that of the healthy mucosa (*p<0.05). Panel B: The contributions of the mucosa and muscularis layers of the colon to H_2_S synthesis via the P5P-dependent pathways. H_2_S production by muscularis and mucosa from sites of ulceration were significantly greater than that from healthy tissue or tissue from non-ulcerated sites (*p<0.05, ***p<0.001). Each bar represents the mean ± SEM of 4–6 rats.

H_2_S synthesis via CAT-3MST from the mucosa and muscularis layers of the colonic tissue from healthy rats was comparable (Figure 5A), as was the case for H_2_S synthesis via P5P-dependent pathways (Fig. 5B). However, the mucosa from sites of ulceration produced significantly more H_2_S via the CAT-3MST pathway (Fig. 5A) and the P5P-dependent pathways (Fig. 5B). H_2_S synthesis from samples of the muscularis layer were relatively low, though a significant increase in synthesis via the P5P-dependent pathways was observed when the tissue was from a region underling a site of ulceration (Fig. 5B). H_2_S synthesis from mucosal or muscularis samples from rats with colitis, but taken from sites that were not ulcerated, was similar to that from samples taken from healthy controls. However, the elevated synthesis of H_2_S from tissues taken from sites of ulceration did not appear to be related to inflammation (e.g., granulocyte infiltration) at those sites. MPO activity, a marker of granulocyte infiltration, was comparable in samples of mucosa from sites of ulceration versus non-ulcerated sites (21.5±0.5 U/mg versus 17.8±2.0 U/mg, respectively; ns), but significantly elevated versus healthy controls (5.1±0.5 U/mg; p<0.05). The same was the case for samples of muscularis from sites of ulceration versus non-ulcerated sites (10.9±2.9 U/mg vs. 8.6±3.2 U/mg, respectively; ns), in contrast to the significant elevation of MPO versus healthy controls (0.5±0.2 U/mg; p<0.05).

### Increased expression of H_2_S-producing enzymes during colitis

**Figure 6:**
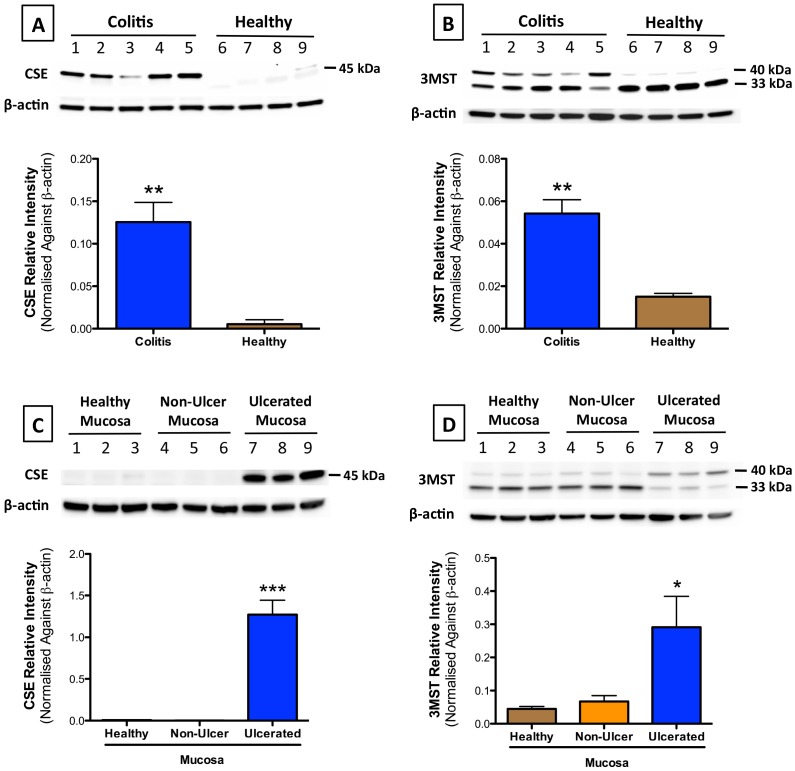
Expression of 3MST and CSE in the healthy and damaged colon. Each panel includes Western blots with an associated analysis of relative intensity of enzyme expression, normalized to β-actin expression. Panels A and B: The expression of CSE (A) and 3MST (B) were markedly increased in the colonic tissue from rats with colitis as compared to tissue from healthy controls (**p<0.01). Panels C and D: The expression of CSE (C) and 3MST (D) were significantly increased at sites of mucosal ulceration as compared to adjacent, non-ulcerated tissue and to tissue from healthy controls (***p<0.001 and *p<0.05, respectively). Each bar represents the mean ± SEM of 3–5 rats.

Consistent with the predominance of CSE as a source of P5P-dependent H_2_S synthesis, expression of this enzyme was markedly up-regulated in inflamed colonic tissue as compared to tissue from healthy controls (Fig. 6A). Likewise, Western blots confirmed the presence of CAT (data not shown) and 3MST in the healthy and inflamed colon of rats (Fig. 6B), with a substantial increase in 3MST expression in inflamed colonic tissue (Fig. 6B). Interestingly, the increased expression of 3MST and CSE during colitis occurred at sites of mucosal ulceration, but not in non-ulcerated tissue from rats with colitis or in tissue from healthy controls (Fig. 6C and D).

### H_2_S degradation is markedly reduced at sites of ulceration

**Figure 7:**
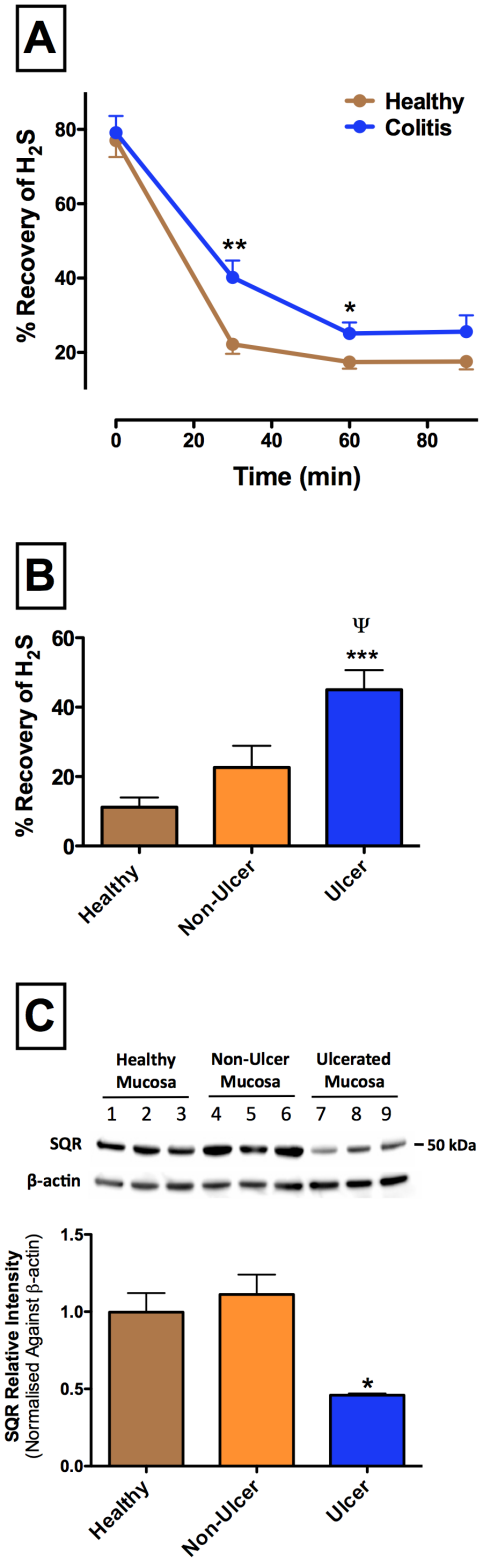
Catabolism of H_2_S by healthy and ulcerated colonic tissue. Panel A: Catabolism of H_2_S was significantly reduced in tissue from rats with colitis as compared tissue from healthy controls (*p<0.05, **p<0.01). Each point represents the mean ± SEM of 5–10 samples. Panel B: Catabolism of H_2_S was significantly reduced in mucosal tissue from sites of ulceration as compared to mucosal tissue from healthy controls (***p<0.001) and as compared to mucosal tissue from adjacent, non-ulcerated sites in rats with colitis (^ψ^p<0.05). Recovery of H_2_S was measured 30 min after addition of NaHS to the tissue samples. Panel C: Expression of sulfide quinone reductase (SQR) was significantly reduced (*p<0.05) at sites of ulceration as compared to adjacent, non-ulcerated mucosa and to tissue from healthy controls. Each bar represents the mean ± SEM of 5 rats.

Both the healthy and inflamed colon rapidly inactivated H_2_S *in vitro* (Figure 7A), but there was significantly less inactivation of H_2_S in samples from rats with colitis than in healthy controls. Approximately 20% of the H_2_S that was added to the samples could not be recovered, and presumably was bound to tissue. Indeed, pretreatment of the tissue samples with potassium cyanide (1 mM) increased the recovery of H_2_S after 30 min of incubation substantially (to 56.7+5.6% for tissue from healthy rats, and to 63.6+4.6% for tissue from rats with colitis). This suggests that the majority of the decrease in recovery of H_2_S from the tissue samples was due to metabolic inactivation.

Inactivation of H_2_S was substantially reduced in mucosal samples taken from sites of ulceration than that from adjacent, non-ulcerated sites or from samples from healthy controls (Figure 7B). This reduced ability to inactivate H_2_S coincided with a significant reduction of the expression of SQR at sites of mucosal ulceration when compared to immediately adjacent, non-ulcerated sites or to healthy controls (Figure 7C). No differences in inactivation of H_2_S were observed for muscularis samples from healthy rats or rats with colitis, and there were no differences in H_2_S recovery between muscularis samples taken from sites of ulcerated versus non-ulcerated sites (data not shown).

## Discussion

H_2_S is produced throughout the GI tract [47], [48] and contributes to many digestive functions [49], including epithelial secretion [20]–[22], smooth muscle contraction [18], [19] and mucosal defense [14], [23]–[26], [50]. H_2_S also promotes resolution of inflammation and repair of injury in the gastrointestinal tract and elsewhere [7], [14], [25], [26], [51]. In the present study we have demonstrated that colonic H_2_S synthesis does not occur solely through P5P-dependent pathways (CSE and CBS). Rather, a P5P-independent, α-KG-dependent pathway via the enzymes CAT and 3MST is the major source of H_2_S synthesis in both the healthy and inflamed colon. We also demonstrated that in colitis the ulcerated mucosa is the major site of both P5P-dependent and α-KG-dependent H_2_S synthesis and that this synthesis is not influenced by the extent of granulocyte infiltration into the tissue. Furthermore, inactivation of H_2_S occurs rapidly in colonic tissue (in the mucosa much more quickly than in muscle), but at a significantly lower rate in ulcerated colonic tissue than in tissue immediately adjacent to ulcers or in healthy colonic tissue. Moreover, increased expression of H_2_S-producing enzymes and decreased expression of the key H_2_S inactivating enzyme (SQR) were observed specifically at sites of mucosal ulceration, but not in mucosa immediately adjacent to ulcers or in the healthy mucosa. One explanation for the decrease in expression of SQR at sites of ulceration is that it is just a consequence of the tissue destruction at those sites. However, at these same sites we observed significant up-regulation of 3MST, which like SQR, is primarily localized to mitochondria.

Our observation that H_2_S synthesis was selectively up-regulated at sites of ulceration is consistent with an important role of this mediator in driving tissue repair [25], [27], [52]. We previously observed a marked up-regulation of CSE and CBS expression and a corresponding increase in H_2_S synthesis at the margins of gastric ulcers, which is the key area of re-epithelialization and angiogenesis that drive ulcer healing [27]. Administration of inhibitors of H_2_S synthesis resulted in delayed ulcer healing, while administration of H_2_S donors accelerated ulcer healing [27]. The healing of ulcers in the colon has similarly been shown to be enhanced by H_2_S donors [10], [25], [28], [29]. It is well recognized that enzymes producing other mediators that promote ulcer healing, such as cyclooxygenase-2 and endothelial nitric oxide synthase, are also up-regulated at the margins of ulcers [53], [54]. The trigger(s) for the elevated H_2_S synthesis at sites of ulceration are not yet known, but one possibility is that local release of vascular endothelial growth factor could play an important role. Vascular endothelial growth factor is an important signal for ulcer healing in the gastrointestinal tract and has been shown to promote angiogenesis, a crucial step in ulcer healing [55], in part through induction of CSE and elevation of H_2_S synthesis [52]. Bacteria-derived signals may also be involved in triggering up-regulation of colonic H_2_S synthesis when there are breaches of the epithelial barrier.

The levels of H_2_S at any given site is dependent upon rates of synthesis and of catabolism. Thus, the reduced rates of inactivation of H_2_S that we observed at sites of ulceration would contribute to producing elevated local levels of H_2_S, further contributing to healing. In healthy colonic tissue H_2_S is rapidly degraded, mainly by oxidation to thiosulfate, and primarily through the enzymes SQR and rhodanese (thiosulfate transferase) [30], [51], [56]–[58]. SQR and rhodanese are highly expressed in the healthy colonic epithelium [30], [51], [56], [59]. In vitro, colonic epithelial cells are capable of oxidizing H_2_S up to concentrations of 50 uM, via the mitochondrial sulfide oxidizing enzymes (59). The reduced inactivation of H_2_S at sites of ulceration may be attributable to the observed, marked reduction of SQR expression specifically at sites of ulceration in the colon. Linden et al. [58] recently suggested that these efficient catabolic pathways act to keep resting levels of H_2_S in tissue low, thereby enhancing the ‘on demand’ signaling capacity of this molecule. We would speculate that in a circumstance of impaired epithelial barrier function, it is possible that H_2_S derived from luminal bacteria may also influence repair processes, and bacterial products such as endotoxin and butyrate may be a further stimulus for induction of H_2_S-synthesizing enzymes at sites of injury [60], [61]. Consistent with this hypothesis, Shen et al. recently reported that germ-free mice have a decreased bioavailability of H_2_S in a range of tissues, including the colon, when compared to mice raised in a specific pathogen-free environment [62]. The mechanism underlying this observation remains unknown.

Our observation that most of the H_2_S produced by colonic tissue is derived via a α-KG-dependent pathway was surprising. Previous studies have focused on the enzymes CSE and CBS as significant sources of H_2_S synthesis both in healthy and inflamed colonic tissue [25], [28], [48]. We previously reported that CBS was the major enzymatic source of colonic H_2_S, but this was based largely on our observation that CHH, an inhibitor of CBS, caused a dramatic exacerbation of colitis in rats, that was more profound than was seen with inhibitors of CSE [25]. However, in the present study, using CSE−/− and CBS+/− mice, we found that CSE was the major source of P5P-dependent H_2_S synthesis in the healthy colon. Moreover, while CHH has been widely used as an inhibitor of CBS and has been shown to suppress P5P-dependent H_2_S synthesis [23], [48], [63], it also inhibits aminotransferases [62], [64], and therefore can block the synthesis of H_2_S that occurs via the CAT-3MST pathway. This was confirmed in the present study. A recent study by Asimakopoulou et al. [65] tested the selectivity of several commonly used putative inhibitors of H_2_S-producing enzymes and concluded that no selective inhibitors of CBS are currently available.

In summary, the CAT-3MST enzymes represent the primary pathway for H_2_S synthesis in the healthy and inflamed colon. Marked increases in colonic H_2_S synthesis, via both P5P-dependent and α-KG-dependent pathways, occur specifically at sites of ulceration. The signals responsible to triggering this up-regulation of H_2_S synthesis are not yet clear. A marked reduction in tissue inactivation of H_2_S also occurs selectively at sites of ulceration. The resulting higher local levels of H_2_S in the microenvironment of the ulcer would act to promote rapid restoration of epithelial barrier integrity. These findings may have important implications with respect to improving treatments for chronic ulcerative conditions of the gastrointestinal tract, including Crohn's disease and ulcerative colitis.
